# Supplementary material for: MetaRibo-Seq measures translation in microbiomes
Source: Nat Commun. 2020 Jun 29;11:3268. doi: 10.1038/s41467-020-17081-z (PMC7324362; doi:10.1038/s41467-020-17081-z)
Supplement: Supplementary file 10 — Supplementary Data 7 [file 41467_2020_17081_MOESM10_ESM.zip › File2/Confidence_VeryHigh_Taxonomy/84801_out.krona.html]

Javascript must be enabled to view this page.

members
magnitude
magnitudeUnassigned
count
unassigned
taxon
rank

84801\_out

9

9
2
superkingdom

9
976
phylum

class
9
200643

171549
9
order

9
171552
family

genus
1
577309

454154
1
species

SRS016541\_contig\_number\_contig-100\_6036.79573

species
370804
5

SRS013687\_contig\_number\_23985SRS013800\_contig\_number\_19928SRS023914\_contig\_number\_22438SRS024435\_contig\_number\_40794SRS1041143\_contig\_number\_1421

genus
3
838

species
1262937
1

SRS049446\_contig\_number\_10834


SRS049959\_contig\_number\_36189SRS149325\_contig\_number\_8389
2
1947102
species
